# Maternal consumption of caffeine and second-hand tobacco smoke as risk factors for the development of oral clefts

**DOI:** 10.1016/j.clinsp.2023.100266

**Published:** 2023-08-09

**Authors:** Rodrigo Soares de Andrade, Fabrício Emanuel Soares de Oliveira, Daniella Reis Barbosa Martelli, Letízia Monteiro de Barros, Hercílio Martelli Júnior

**Affiliations:** a Postgraduate Program in Stomatology, FOP-UNICAMP, Piracicaba, São Paulo, Brazil; b Postgraduate Program in Primary Health Care, Unimontes, Montes Claros, Minas Gerais, Brazil; c Postgraduate Program in Health Sciences, Unimontes, Montes Claros, Minas Gerais, Brazil; d José do Rosário Vellano University, Institute of Dentistry and Health Sciences, Periodontics, Alfenas, Minas Gerais, Brazil

**Keywords:** Caffeine, Tobacco, Cleft palate, Cleft lip, Alcohol consumption

## Abstract

**Objective:**

The aim of this case-control study was to investigate environmental factors, such as caffeine, folic acid, nutritional iron supplementation, multivitamin complexes, alcohol, and tobacco (second-hand smoking), which have been described as risk factors for the development of oral clefts.

**Methods:**

This case-control study employed convenience sampling and included 409 mothers: 132 with children with oral clefts (cases) and 277 with children without oral clefts (controls). The age range of the children in both groups was 0 to 2 years. A questionnaire was administered to each mother to inquire about their habits and food consumption during the first trimester of pregnancy.

**Results:**

Folic acid supplementation was observed in 116 (87.8%) of the case group (*p* < 0.001) and 271 (97.8%) of the control group. Regarding the use of ferrous sulfate, 114 (86.3%) of the case group and 271 (97.8%) of the control group reported using it. In the case group, 84 (63.6%) mothers reported being exposed to second-hand smoke, and 5 (3.7%) reported alcohol consumption (*p* = 0.797). In terms of caffeine consumption, 127 mothers (95.4%) in the case group consumed it (*p* = 0.13), while 247 (88.8%) reported consumption in the control group.

**Conclusions:**

The results suggest a direct relationship between secondhand smoke, alcohol consumption, and the lack of maternal supplementation with oral clefts.

## Highlights

• Several environmental and genetic factors may cause oral clefts.
• Iron supplementation use of folic acid are protective factors for oral fissures.
• Passive smoking during pregnancy can be a risk factor for oral clefts.

## Introduction

Oral clefts are among the most prevalent congenital abnormalities in humans^1^ and have a significant social impact, leading to substantial morbidity and mortality worldwide.^2^ In 70% of cases, Cleft Lip and/or Palate present as Non-Syndromic (NSCL/P), meaning they occur without structural malformations in other organs or behavioral and cognitive alterations.^3^^,^^4^ Based on epidemiological features and embryologic timing, NSCL/P are traditionally classified into Cleft Lip (NSCL), Cleft Lip and Palate (NSCLP), and Cleft Palate (NSCP).^5^

The incidence of NSCL/P is approximately 1 in 500‒2,000 live births, varying according to the geographical location, ethnicity, and socioeconomic status of the population studied.^1^ In Brazil, studies on the incidence of NSCL/P are scarce and show considerable variation. According to the Brazilian surveys, the incidence of NSCL/P ranges from 0.19 to 1.54 for every 1,000 live births.^6^^,^^7^

The etiology of NSCL/P is attributed to interplay of genetic, epigenetic, and environmental factors, but the exact interactions are poorly understood.^5^^,^^8^ This lack of knowledge is likely a reflection of the wide diversity of molecular mechanisms related to facial embryogenesis, involving multiple genes and the influence of environmental factors.^9^, ^10^, ^11^, ^12^ Among the major environmental risk factors for the development of NSCL/P are drug use, alcohol consumption, smoking, maternal diet, and vitamin supplementation, particularly during the first trimester of pregnancy.^13^, ^14^, ^15^

The objective of this control case study was to investigate a correlation to environmental factors, such as caffeine, folic acid, multivitamin complexes, alcohol, and tobacco (second-hand tobacco smoke), which were described as susceptibility to NSCL/P in delimited populations, verify whether there is a correlation between the studied factors and oral cleft.

## Material and methods

This case-control study, utilizing convenience sampling, was conducted at the “Pro-Sorriso” Head and Neck Anomaly Treatment Center in Alfenas, Minas Gerais, Brazil, following the STROBE guidelines. A total of 132 mothers with children up to 24 months of age with NSCL/P were recruited for the case group. Additionally, 277 mothers with children without craniofacial changes, who were being followed at the Alzira Vellano Hospital in Alfenas, Minas Gerais, Brazil, were randomly selected for the control group. The sample was chosen based on convenience, meaning all patients during the study's specified time period were included.

The sample of cases included mothers whose children had newly diagnosed NSCL/P and were younger than 24 months, without distinction of sex. The controls were selected using the same method but consisted of children without any skull alterations or syndromes. Each control was matched with a case of the same gender and similar age within a 3-month range. All children with oral clefts underwent evaluation by a multidisciplinary team of specialists to exclude the presence of other alterations or syndromes.

The sample of the studied population was selected through convenience sampling over the course of one year of follow-up. The selection was based on the primary exposures of interest, which included second-hand tobacco smoke, supplementation with folic acid, ferrous sulfate, and multivitamin complexes, as well as caffeine and alcohol consumption during the first trimester of pregnancy.

The mothers completed a demographic questionnaire regarding their characteristics, habits, and exposures during pregnancy. The questionnaire included information on consanguinity, history of NSCL/P, use of folic acid, consumption of an iron-rich diet, use of vitamins and ferrous sulfate, exposure to second-hand tobacco smoke, alcohol consumption, and consumption of coffee. The questionnaire included questions about maternal consumption of caffeine-containing beverages, such as coffee, caffeinated soft drinks, energy drinks, and dietary supplements for physical performance, during the first three months of pregnancy. For each beverage, there was one question with five response categories: none, number of 100 mL cups per week on average (without specifying cup size).^13^, ^14^, ^15^

The questionnaire was administered to all mothers following a standardized method, focusing on their food, nutritional supplementation, and habits during pregnancy, with particular emphasis on the first trimester.

To analyze the beverage consumption, all reported beverages were treated in the same manner. The variable “cups per week” was calculated by converting the reported number of cups consumed per day to a weekly basis (by dividing or multiplying by 7). Women who reported consuming less than 1 cup per week were categorized as having zero consumption.

The variable “concentration in milligrams per week” was treated as a continuous variable. The caffeine content of each beverage was estimated as follows: 100 mg per cup of coffee, 40 mg per cup of caffeinated soft drinks, 80 mg per cup of dietary supplements for physical performance, and 70 mg per thermogenic capsule. These values were based on information provided by international health authorities, specifically from the webpage (http://www.matportalen.no/Emner/Gravide). The variable “concentration in milligrams per week” was then categorized into two groups: consumption ranging from 100 mg to 500 mg per week and consumption greater than 500 mg per week.

All analyses were conducted using IBM SPSS Statistics (version 20.0; SPSS Inc., Chicago, Illinois, USA) with a significance level of 5%. Descriptive analyses were performed for categorical variables, reporting frequencies and percentages. The bivariate analysis utilized the chi-square test to examine the relationship between the exposures of interest and oral clefts.

For multivariate analysis, logistic regression was conducted to calculate adjusted odds ratios with 95% Confidence Intervals and p-values. The feasibility of the multivariate analysis was assessed using the multicollinearity test, and any variables demonstrating collinearity were excluded. Additionally, variables that were found to be significant risk factors in the chi-square test were included in the multivariate analysis. The “stepwise backward” method was employed for the multivariate logistic regression. The goodness of fit of the model was assessed using the Hosmer and Lemeshow test.

The study fulfilled the ethical requirements for conducting research involving human subjects and received approval from the institutional Research Ethics Committee (n° 87322318.2.3001.5418).

## Results

Five (3.8%) mothers from the case group, who had children with oral clefts, and three from the control group (1.10%) reported consanguinity in their children due to being married to first-degree relatives. However, this difference was not statistically significant (*p* = 0.065). In the case group, 18 mothers (13.64%) reported a history of oral cleft in the child's maternal or paternal family. When the authors observed folic acid supplementation in the first trimester of pregnancy, 116 (87.88%) (*p* < 0.001) mothers of the case group and 271 (97.83%) of the control group used correctly (OR = 6.23; 95% CI 2.37‒16.32; *p* < 0.001) (Table 1).

**Table 1:** Values referring to the variables evaluated in this study, which may have a direct relationship with the development of oral clefts.

		Case	Control	p-value a	OR ratio (95%)
		n = 132 (%)	n = 277 (%)		
Consanguinity	No	127 (96.2)	274 (98.9)	0.065	Reference
	Yes	5 (3.8)	3 (1.1)		3.59 (0.84‒15.27)
NSCL/P history	No	114 (86.3)	270 (97.4)	<0.001	Reference
	Yes	18 (13.6)	7(2.5)		1.18 (1.09‒1.28)
Folic acid	Yes	116 (87.8)	271 (97.8)	<0.001	Reference
	No	16 (12.1)	6 (2.1)		6.23 (2.37‒16.32)
Nutritional supplementation (iron)	Yes	109 (82.5)	223 (80.5)	0.617	Reference
	No	23 (17.4)	54 (19.5)		0.87 (0.50‒1.49)
Vitamin and ferrous sulfate supplementation	Yes	114 (86.3)	271 (97.8)	<0.001	Reference
	No	18 (13.6)	6 (2.1)		7.13 (2.75‒18.43)
Second-hand tobacco smoke > 15 min	No	48 (36.3)	218 (78.7)	<0.001	Reference
	Yes	84 (63.6)	59 (21.3)		6.46 (4.09‒10.20)
Alcohol consumption	No	127 (96.2)	265 (95.6)	0.797	Reference
	Yes	5 (3.7)	12 (4.3)		0.86 (0.30‒2.52)
Caffeine consumption	No	5 (3.7)	31 (11.1)	0.013	Reference
	Yes	127 (96.2)	247 (88.8)		3.20 (1.21‒8.43)

a Chi-square test.

Regarding iron supplementation, 109 (82.57%) (*p* = 0.617) of the mothers in the case group reported making dietary changes in during the first trimester of pregnancy, opting for healthier foods rich in vitamins and iron such as vegetables, fruits, and iron-rich foods. Similarly, 223 (80.50%) (OR = 0.87; 95% CI 0.50‒1.49; *p* = 0.617) mothers in the control group also mentioned improving their dietary patterns. When questioned about the use of pre-gestational vitamins and ferrous sulfate, 114 (86.37%) mothers in the case group reported supplementation, while 271 (97.83%) mothers in the control group also used them. The odds ratio was 7.13 (95% CI 2.75‒18.43; *p* < 0.001), indicating a significantly higher prevalence of vitamin and ferrous sulfate supplementation in the control group (Table 1).

Regarding deleterious habits such as second-hand tobacco smoke and alcohol consumption during the first trimester of pregnancy, 84 (63.63%) mothers in the case group reported being exposed to second-hand tobacco smoke (OR = 6.46; 95% CI 4.09‒10.20; *p* < 0.001). Additionally, 5 (3.78%) mothers admitted to frequent alcohol use, but the p-value was not significant (OR = 0.86; 95% CI 0.30‒2.52; *p* = 0.797). In the control group, 59 (21.29%) mothers reported being exposed to second-hand tobacco smoke, while 12 (4.33%) mothers admitted to consuming alcohol during the first trimester of pregnancy (Table 1). In the case group, 127 mothers (96.2%) reported frequent consumption of caffeine-containing products during pregnancy (*p* = 0.013). In the control group, out of the 277 mothers, 247 (88.8%) reported consumption (OR = 3.20; 95% CI 1.21‒8.43; *p* = 0.013) (Table 1).

Of the 127 mothers in the case group who reported caffeine use, 107 (84.3%) mothers reported consumption of more than 500 mg per week, and of the 247 mothers in the case group, 204 (82.46%) consumed more than 500 mg weekly. When the authors compared caffeine intake by a group of mothers, the authors observed that the consumption of more than 500 mg of caffeine was similar for mothers of patients in both groups (OR = 1.12; 95% CI 0.63–2.01; *p* = 0.684) (Table 2).

**Table 2:** Reference values of caffeine consumption of the case and control groups.

	Case	Control	p-value^a^	OR ratio (95%)
	n = 127 (96.2%)	n = 247 (88.8%)		
100 ≤ 500 mg	20 (15.7%)	43 (17.4%)		Reference
> 500 mg	107 (84.3%)	204 (82.6%)	0.684	1.12 (0.63‒2.01)

a Chi-square test.

In the bivariate chi-square analysis, there was a significant association between the risk of NSCL/P and coffee consumption (OR=3.20; 95% CI 1.21‒8.43; *p* = 0.013). However, when adjusted by factors such as folic acid history, vitamin and ferrous sulfate supplementation, and second-hand tobacco smoke, coffee consumption did not show a significant association in the multivariate logistic regression. In the final logistic regression model, it was observed that the consumption of iron-rich foods (*p* = 0.007) and the supplementation of vitamins and ferrous sulfate (*p* < 0.001) during pregnancy were identified as protective factors against the development of oral clefts. On the other hand, second-hand tobacco smoke exposure during pregnancy was found to be a significant risk factor (*p* < 0.001), increasing the chances of developing oral clefts by 5.62 times (Table 3).

**Table 3:** Result of multiple logistic regression. Variables that remained in the final model with significance.

Variables	p-value a	OD ratio (95%)
Nutritional supplementation (iron)	0.007	0.22 (0.07‒0.66)
Vitamin and ferrous sulfate supplementation	<0.001	0.03 (0.08‒0.17)
Second-hand tobacco smoke > 15 min	<0.001	5.62 (3.26‒9.67)

a Multivariate logistic regression.

## Discussion

Some studies have shown that the habits of mothers during the gestational period are directly associated with a higher risk of developing oral clefts.^15^^,^^16^ In this study, the authors focused on mothers receiving prenatal care and analyzed their consumption of dietary supplements that are known to promote proper fetal development, such as folic acid and ferrous sulfate.^17^ These factors are primarily associated with the fusion and development of the maxillary processes. The findings of this study are in line with the findings of Taghavi et al., who similarly reported that mothers of children without oral clefts tend to receive proper prenatal care and exhibit better supplementation practices compared to mothers of children with oral clefts.^18^

Second-hand tobacco smoke is considered deleterious during pregnancy, as established by the World Health Organization (WHO) in 2003 (https://www.who.int/publications/i/item/9789241506076). The precise mechanisms by which tobacco increases the risk of craniofacial abnormalities in infants are not fully understood. However, the most likely explanation is the interaction between smoking and polymorphism of susceptible genes.^19^

The inhaled CO_2_ undergoes dissociation within the mother's body, releasing free radicals that can have teratogenic effects. Additionally, the vasoconstrictive action of nicotine can lead to a reduction in uteroplacental blood flow. Carbon monoxide binds to hemoglobin, reducing the availability of oxygen to the placenta.^20^ Furthermore, tobacco-induced endothelial injury increases the likelihood of placental neovessel rupture, leading to a decrease in fetal blood supply and resulting in hypoxia. This hypoxia is believed to contribute to abnormal facial morphogenesis. Therefore, the combined effects of toxin exposure, hypoxia, and cellular ischemia contribute to the development of craniofacial abnormalities.^19^ Little et al^20^ conducted an analysis involving 24 mothers to investigate the impact of smoking during pregnancy on the occurrence of oral clefts in their children. The study revealed a statistically significant association between maternal smoking and NSCL/P, with a relative risk of 1.34. This finding supports the evidence from the current study, where a higher proportion of mothers with children with clefts reported exposure to smoking compared to mothers in the control group. It indicates a potential predisposition for mothers whose children have clefts when exposed to smoke, and this variable maintained its statistical significance in the multiple analysis, highlighting its consistent association with the development of oral clefts.^21^

There is no exact mechanism by which coffee ingestion can increase the risk of oral clefts,^14^ it is believed that the effects on homocysteine levels may play a role. There is evidence suggesting a link between maternal hyperhomocysteinemia and an increased risk of clefts.^16^ Both coffee ingestion^21^ and exposure to second-hand tobacco smoke^22^ have been shown to increase the plasma concentration of homocysteine. Additionally, these factors have been associated with an elevated risk of spontaneous abortions, chromosomal anomalies, multiple congenital aberrations,^23^ and notably, low birth weight.^24^

Supplementation with folic acid and ferrous sulfate is commonly associated with a reduction in the risk of oral clefts.^25^ These supplements also help in reducing plasma homocysteine levels.^21^ which further justifies their use during the pre-gestational period. Maintaining an iron-rich diet and taking vitamins and ferrous sulfate during pregnancy were identified as protective factors, associated with a lower likelihood of oral clefts. These findings were statistically significant in the final regression model.

Regarding alcohol consumption, there was no statistical significance observed in this study, although there is strong evidence indicating an association between alcohol use during pregnancy and the occurrence of oral clefts.^26^ One possible explanation for the lack of association in this particular study could be the small number of participants who reported alcohol consumption during pregnancy in both the case and control groups.^27^

Considering that the fusion of the maxillary processes takes place relatively late in the first trimester,^28^ it is possible that the reported coffee intake may be higher than the actual consumption during the critical phase of embryonic development. This could be attributed to the fact that over time, coffee intake tends to decrease due to symptoms like nausea and vomiting experienced by pregnant women. A meta-analysis of three studies on maternal coffee consumption and oral clefts revealed a slight increase in the risk of clefts,^29^ which supports the present study's findings of an association between caffeine consumption and the likelihood of clefts developing in the bivariate analysis. In the study conducted by Kurppa et al.,^30^ drinking more than 4 cups of coffee per day was not associated with an increased risk of oral clefts (OR = 1.0; 95% CI 0.6‒1.6). However, in the study by Qian et al.,^31^ consuming more than 3 cups of coffee per day resulted in an adjusted odds ratio of 1.4 (95% CI 0.7‒2.7).^29^ In the study by Billie et al.,^32^ which included 134 mothers, caffeine consumption was associated with an increased risk of NSCL/P, further supporting the findings of this study.

Concentration parameters of caffeine in different sources, portion sizes, and preparation methods for coffee should be taken into account when estimating caffeine intake. However, the methodology^13^, ^14^, ^15^ used for this estimation has been extensively validated in a subpopulation, utilizing various biomarkers and reference measures. The agreement between the food diaries was notably high for coffee and cola-based soft drinks, which are the main sources of caffeine in the studied population.^33^^,^^34^ The knowledge of teratogenic factors is important for every health professional as it is their responsibility to monitor and provide guidance to women during pregnancy. Clinically, it is crucial to be aware of these etiologic factors in order to provide accurate guidance and proper monitoring throughout pregnancy, leading to the reduction of risks associated with the development of craniofacial anomalies.^35^

This study presents some limitations due to the time of development and how much consumption measures are reported, which may be different for each person. Convenience sampling also limits the possibility of inferences from the study results.

## Conclusion

Passive smoking and alcohol consumption during the first trimester of pregnancy, along with inadequate prenatal care, increase the risk of oral clefts in children. From a clinical perspective, it is crucial to be aware of these etiologic factors in order to provide accurate guidance and appropriate prenatal care, ultimately minimizing the risks associated with the development of craniofacial anomalies.

## Clinical Trial registration number

As it is a case-control study, this work does not have a clinical trial registration number.

## CRediT authorship contribution statement

**Rodrigo Soares de Andrade:** Conceptualization, Data curation, Formal analysis, Investigation, Methodology, Writing – original draft, Writing – review & editing. **Fabrício Emanuel Soares de Oliveira:** Conceptualization, Formal analysis, Methodology, Writing – review & editing. **Daniella Reis Barbosa Martelli:** Conceptualization, Formal analysis, Methodology, Project administration, Writing – review & editing. **Letízia Monteiro de Barros:** Conceptualization, Data curation, Investigation, Methodology, Validation, Writing – review & editing. **Hercílio Martelli Júnior:** Conceptualization, Formal analysis, Investigation, Methodology, Supervision, Validation, Visualization, Writing – original draft, Writing – review & editing.

## Declaration of Competing Interest

The authors declare no conflicts of interest.

## References

[1] Machado RA, Popoff DAV, Martelli-Júnior H. (2022). Relationship between non-syndromic oral clefts and cancer: a systematic review and meta-analysis. Oral Dis.

[2] Asllanaj B, Kragt L, Voshol I, Koudstaal M, Kuijpers MA, Xi T (2017). Dentition patterns in different unilateral cleft lip subphenotypes. J Dent Res.

[3] Schutte BC, Murray JC. (1999). The many faces and factors of orofacial clefts. Hum Mol Genet.

[4] Alade A, Awotoye W, Butali A. (2022). Genetic and epigenetic studies in non-syndromic oral clefts. Oral Dis.

[5] Machado RA, de Freitas EM, de Aquino SN, Martelli DRB, Swerts MSO, Reis SRA (2017). Clinical relevance of breast and gastric cancer-associated polymorphisms as potential susceptibility markers for oral clefts in the Brazilian population. BMC Med Genet.

[6] Martelli-Junior H, Porto LV, Martelli DR, Bonan PR, Freitas AB, Della Coletta R (2007). Prevalence of nonsyndromic oral clefts in a reference hospital in the state of Minas Gerais, Brazil, between 2000‒2005. Braz Oral Res.

[7] Rodrigues K, Sena MF, Roncalli AG, Ferreira MA. (2009). Prevalence of orofacial clefts and social factors in Brazil. Braz Oral Res.

[8] Yin B, Shi B, Jia ZL. (2018). Associations among PRDM16 polymorphisms, environmental exposure factors during mother's pregnancy, and nonsyndromic cleft lip with or without cleft palate. Hua Xi Kou Qiang Yi Xue Za Zhi.

[9] Letra A, Menezes R, Granjeiro JM, Vieira AR. (2007). Defining subphenotypes for oral clefts based on dental development. J Dent Res.

[10] Paranaiba LM, Coletta RD, Swerts MS, Quintino RP, de Barros LM, Martelli-Júnior H. (2013). Prevalence of Dental Anomalies in Patients With Nonsyndromic Cleft Lip and/or Palate in a Brazilian Population. Cleft Palate Craniofac J.

[11] Melo Filho MR, Nogueira dos Santos LA, Barbosa Martelli DR (2015). Taurodontism in patients with nonsyndromic cleft lip and palate in a Brazilian population: a case control evaluation with panoramic radiographs. Oral Surg Oral Med Oral Pathol Oral Radiol.

[12] Mangione F, Nguyen L, Foumou N, Bocquet E, Dursun E. (2018). Cleft palate with/without cleft lip in French children: radiographic evaluation of prevalence, location, and coexistence of dental anomalies inside and outside cleft region. Clin Oral Investig.

[13] Johansen AM, Wilcox AJ, Lie RT, Andersen LF, Drevon CA. (2009). Maternal consumption of coffee and caffeine-containing beverages and oral clefts: a population-based case-control study in Norway. Am J Epidemiol.

[14] Dien VHA, McKinney CM, Pisek A, Pitiphat W. (2018). Maternal exposures, and risk of oral clefts in South Vietnam. Birth Defects Res.

[15] Wierzejska R, Jarosz M, Wojda B. (2019). Caffeine Intake during pregnancy and neonatal anthropometric parameters. Nutrients.

[16] Jahanbin A, Shadkam E, Miri HH, Shirazi AS, Abtahi M. (2018). Maternal folic acid supplementation and the risk of oral clefts in offspring. J Craniofac Surg.

[17] Hawkey A, Junaid S, Yao L, Spiera Z, White H, Cauley M (2019). Gestational exposure to nicotine and/or benzo[a]pyrene causes long-lasting neurobehavioral consequences. Birth Defects Res.

[18] Li N, Zhang K, Chen X, Cui J, Han X, Zhai D (2023). Evaluation of growth status of children with non-syndromic oral clefts. J Clin Pediatr Dent.

[19] Lopes GDS, Guimarães L, Nascimento E, Freitas DQ, Rebello I, Medrado AP (2022). Root curvature in non-syndromic oral clefts: a case-control study in a Brazilian population. Cleft Palate Craniofac J.

[20] Little J, Cardy A, Munger RG. (2004). Tobacco smoking and oral clefts: a meta-analysis. Bull WHO;.

[21] Gildestad T, Bjørge T, Vollset SE, Klungsøyr K, Nilsen RM, Haaland ØA (2015). Folic acid supplements and risk for oral clefts in the newborn: a population-based study. Br J Nutr.

[22] Wang P, Wu T, Schwender H, Wang H, Shi B, Wang ZQ (2018). Evidence of interaction between genes in the folate/homocysteine metabolic pathway in controlling risk of non-syndromic oral cleft. Oral Dis.

[23] Crossan E, Duane B. (2018). Is there an association between maternal smoking and oral clefts?. Evid Based Dent.

[24] Yoon NY, Yun I, Park YS, Park EC (2022). Associations between environmental tobacco smoke exposure and oral health symptoms in adolescents. BMC Oral Health.

[25] Hoyt AT, Canfield MA, Romitti PA, Botto LD, Anderka MT, Krikov SV (2016). Associations between maternal periconceptional exposure to secondhand tobacco smoke and major birth defects. Am J Obstet Gynecol.

[26] Coletta RD, Sunavala-Dossabhoy G. (2022). Orofacial clefts: A compendium on non-syndromic cleft lip-cleft palate. Oral Dis.

[27] Yin X, Li J, Li Y, Zou S. (2019). Maternal alcohol consumption and oral clefts: a meta-analysis. Br J Oral Maxillofac Surg.

[28] Lund AE. (2008). First-trimester maternal alcohol consumption may lead to oral clefts. J Am Dent Assoc.

[29] Browne ML. (2006). Maternal exposure to caffeine and risk of congenital anomalies: a systematic review. Epidemiology.

[30] Kurppa K, Holmberg PC, Kuosma E, Saxén L. (1983). Coffee consumption during pregnancy and selected congenital malformations: a nationwide case-control study. Am J Public Health.

[31] Qian J, Chen Q, Ward SM, Duan E, Zhang Y. (2020). Impacts of caffeine during pregnancy. Trends Endocrinol Metab.

[32] Bille C, Olsen J, Vach W, Knudsen VK, Olsen SF, Rasmussen K (2007). Oral clefts and life style factors–a case-cohort study based on prospective Danish data. Eur J Epidemiol.

[33] Zheng Z, Xie G, Yang T, Qin J. (2019). Congenital malformations are associated with secondhand smoke among nonsmoking women: a meta-analysis. Birth.

[34] James JE. (2021). Maternal caffeine consumption and pregnancy outcomes: a narrative review with implications for advice to mothers and mothers-to-be. BMJ Evid Based Med.

[35] Madden LM, Oliva J, Eller A, DiDomizio E, Roosa M, Blanchard L (2022). Pregnant women and opioid use disorder: examining the legal landscape for controlling Women's Reproductive Health. Am J Law Med.

